# Determining the Degree of Perceptible Static Eyelid Asymmetry and Effect of Face Inversion: A Cross-sectional Pilot Study

**DOI:** 10.1097/IOP.0000000000002650

**Published:** 2024-09-05

**Authors:** Frank G. Preston, Ziyaad Sultan, James Hsuan, Kevin J. Hamill, Austin G. McCormick

**Affiliations:** * Department of Eye and Vision Science, Institute of Life Course and Medical Sciences, University of Liverpool; † Department of Ophthalmology, Aintree University Hospital, Liverpool University Hospitals Foundation Trust, Liverpool, United Kingdom

## Abstract

**Purpose:**

To determine the degree of static eyelid asymmetry required to be perceptible and whether this is affected by image inversion.

**Methods:**

Images of 3 volunteers were digitally manipulated to have eyelid asymmetry of 0.5 mm, 1 mm, or 1.5 mm of 3 different types, upper lid ptosis, upper lid retraction, and lower lid retraction. Forty-nine laypersons stated whether the images were symmetrical or asymmetrical. A separate inversion survey, completed by 29 clinicians, consisted of symmetrical images and 1 mm asymmetrical images, with half being inverted.

**Results:**

Upper lid ptosis, upper lid retraction, and lower lid retraction were not detected by most laypeople at 0.5 mm of severity (18.9%, 6.7%, 18.9% detection, respectively) but all 3 were detected by the majority of participants once asymmetry reached 1 mm severity (65.7%, 61.8%, 51.0% detection, respectively) and rose to over 70% identification at 1.5 mm (92.2%, 73.5%, 73.5% detection, respectively). Inversion of the images led to 19.7% lower rates of correct identification of asymmetrical faces compared with images presented in the correct orientation (80.7% asymmetry identified in normal images, 61.0% inverted, *p* < 0.001).

**Conclusions:**

All asymmetries were detectable by a majority of laypersons at a severity of 1 mm. Image inversion decreases a clinician’s ability to detect a 1 mm asymmetry, indicating an impaired asymmetry perception in the intraoperative view. This study provides research to counsel patients with varying degrees of eyelid asymmetry.

## Abstract 2

Lay participants detected a majority of eyelid asymmetries at a 1 mm severity and a clinician’s ability to detect a 1 mm asymmetry is decreased when the image is inverted.

Facial asymmetry arises within the normal growth and development of the face, with minor differences between the 2 halves of the face not affecting esthetic appearance.^1^ Indeed artificially created perfectly symmetrical faces have been found to be unattractive and disconcerting, with a degree of asymmetry being preferable.^2^ However, higher facial symmetry within the normal range of variation is associated with increased attractiveness,^2,3^ with more stark asymmetries being seen as disfigurement.^4^ Clinicians specializing in oculoplastic surgery regularly assess the eyelids for asymmetry. In addition, patients’ requests for treatment, and satisfaction afterwards, are influenced by perceived asymmetry.^5^ However, a paucity of research exists on the degree of eyelid asymmetry required to be perceptible, and how inversion of the face affects this.

Static facial asymmetry is apparent at rest, with eyelid position being the most detectable.^6^ Eyelid asymmetry can be caused by ptosis of the upper eyelid, an abnormally low-lying upper eyelid,^7^ or retraction of the upper eyelid or lower eyelid, when the upper or lower eyelid is drawn back from its normal position.^8^

Facial asymmetry detection is clinically relevant when counseling patients on how perceptible facial asymmetries are to other individuals. This is important as the decision-making process for surgery in these patients is strongly associated with how severely others perceive their disfigurement.^5^ Asymmetries of the eyelids, in addition to functional implications, impact a patient’s psychosocial functioning. Patients with notable ptosis report increased levels of appearance concern, anxiety, and depression in comparison to the general population.^9^

While the decision-making process of treatment planning with severe asymmetry may be relatively straightforward, more “borderline” cases rely to a greater extent on subjective clinical judgement.^10^ Several studies have assessed the perception of static facial asymmetry at varying degrees of severity.^2,10–14^ However, only one study has looked at eyelid asymmetry.^12^ To enable a more informed, evidence-based, and objective clinical judgement on the decision-making process for surgery, the degree of eyelid asymmetry in static facial asymmetry perceptible by laypersons needs to be determined.

During surgery for eyelid asymmetry, the surgeon’s intraoperative view of the face is inverted as the operation is performed from the head of the bed. While many studies have looked at the effect of face inversion on facial identification,^15–17^ no studies have looked at the effect of face inversion on asymmetry perception among clinicians. This is highly clinically relevant because if inverting a face makes it more difficult to assess asymmetry, a surgeon’s judgement may be affected during surgery.

This study aimed to determine the degree of eyelid asymmetry in static facial asymmetry perceptible by laypersons, specifically looking at upper eyelid ptosis, upper eyelid retraction, and lower eyelid retraction. This study also aimed to see what effect face inversion has on the perception of eyelid asymmetry by clinicians.

## MATERIALS AND METHODS

### Facial Photographs and Digital Manipulation

**FIG. 1.**
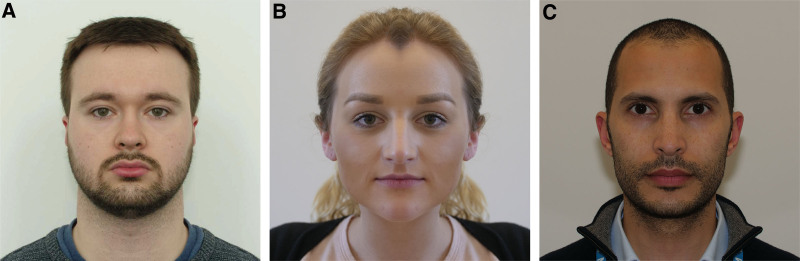
Symmetrical faces. Symmetrical face images of volunteer 1 **(A)**, volunteer 2 **(B)**, and volunteer 3 **(C)**.

High-resolution digital photographs were taken of 3 volunteers in a well-lit environment with a ruler in the same vertical plane as volunteers’ eyelids (Panasonic GH5 camera body with Canon Ef-S 60 mm f2.8 macro lens, Panasonic Corp., Kadoma, Osaka). The images were digitally manipulated to create perfectly symmetrical faces by cropping half of the image and reflecting the remaining half, using Adobe Photoshop (Adobe Corp. San Jose, CA) (Fig. 1). Each of the volunteers gave written consent for the images to be used within the study and for subsequent publication and dissemination.

**FIG. 2.**
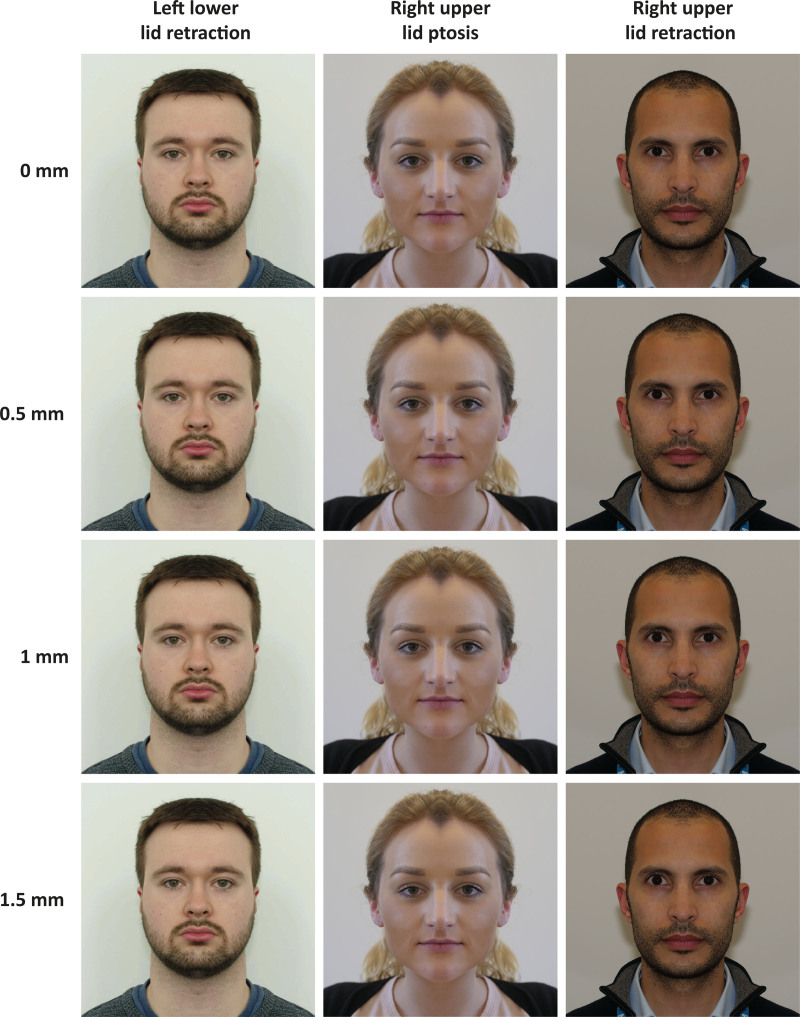
Example eyelid asymmetry images. **Column 1,** left lower lid retraction image series of face 1; **column 2,** right upper lid ptosis image series of face 2; **column 3,** right upper lid retraction. **Row 1,** symmetrical images version; **row 2,** 0.5 mm asymmetry images; **row 3,** 1 mm asymmetry images; **row 4,** 1.5 mm asymmetry images.

The symmetrical images were then digitally manipulated to induce 3 types of eyelid asymmetry (upper eyelid ptosis, upper eyelid retraction, and lower eyelid retraction) at 3 degrees of eyelid asymmetry (0.5 mm, 1 mm, and 1.5 mm) (Fig. 2). Lid levels were manipulated, but lid crease height remained unaltered. This was completed for each of the volunteers’ eyes, meaning each volunteer had a symmetrical image and 6 asymmetrical images (left upper lid ptosis, right upper lid ptosis, left upper lid retraction, right upper lid retraction, left lower lid retraction, and right lower lid retraction) at 0.5 mm, 1 mm, and 1.5 mm degrees of severity. The ruler in the same vertical plane as volunteers’ eyelids within the image was used for measuring asymmetry size.

### Surveys

Three surveys were created to test the degree of eyelid asymmetry required to be perceptible by laypersons (university students and staff). Each survey contained 3 symmetrical images and 6 asymmetrical images of each of the 3 volunteers, resulting in 27 images per survey. In the first survey, the “0.5 mm survey,” all eyelid asymmetries were of 0.5 mm; in the second survey, the “1 mm survey,” all eyelid asymmetries were of 1 mm; and in the third survey, the “1.5 mm survey,” all eyelid asymmetries were of 1.5 mm.

A fourth survey, the “inversion survey,” was created to test the effect of image inversion on the detection of eyelid asymmetries by clinicians. The survey contained 3 symmetrical images and 6 asymmetrical images of 2 volunteers, and the same images again but inverted by rotating the image 180°. This resulted in a total of 36 images.

Facial images were viewed by participants using Jisc Online Surveys (Jisc, Redcliffe, Bristol). Participants of the 0.5 mm, 1 mm, and 1.5 mm surveys were first shown example symmetrical images for each volunteer face. The 27 test images were then presented in a random order. Participants of the inversion survey were shown example symmetrical images for both volunteer faces, and then presented with the 36 test images in a random order.

In all surveys, the participants were instructed to state whether they thought the face was symmetrical or asymmetrical.

### Participants

Participants of the 0.5 mm, 1 mm, and 1.5 mm surveys were students or staff at the University of Liverpool. Participants of the inversion survey were either consultant, specialty doctors, trainees, or allied health care professionals. Student and staff participants were recruited through advertisement within the department weekly email. Clinical staffs were recruited through advertisement in an email sent to a local ophthalmology trainees email list and the British Oculoplastic Surgery Society email list. All participants gave informed written consent to take part in the study and the data were collected and stored in accordance with the Data Protection Act (2018) and the study was conducted in accordance with the Declaration of Helsinki. Volunteers, whose faces were photographed for the surveys, gave consent for the publication of identifiable photographs. Ethical approval was obtained within the university before the participants were invited to take part (ethics approval number: 2593).

### Data Analysis

IBM SPSS Statistics 27.0.1.0 (SPSS Inc., Chicago, IL) was used to perform all statistical analysis on the data. Descriptive statistics were performed on all the data collected. A Pearson χ^2^ test was used to test statistical differences between the number of faces correctly identified as symmetrical/asymmetrical in the 0.5 mm survey, 1 mm survey, 1.5 mm survey, and inversion survey. The statistical significance used for all analysis performed was *p* < 0.05.

## RESULTS

A total of 49 lay participants took part in the asymmetry perception surveys. The participant demographics were similar across the 0.5 mm, 1 mm, and 1.5 mm surveys (Table 1). Fifteen, seventeen, and seventeen participants undertook the 0.5 mm, 1 mm, and 1.5 mm survey, respectively, looking at 27 faces each, resulting in a total of 405, 459, and 459 observations being counted, respectively (Table 2).

**TABLE 1.** Demographics of asymmetry perception survey participants

	0.5 mm survey	1 mm survey	1.5 mm survey	Inversion survey
n	15	17	17	29
Age (years)	29.5 ± 11.5	34.6 ± 14.0	29.5 ± 10.3	46.7 ± 10.7
Gender (female) (%)	73.3%	47.1%	76.5%	41.4%

**TABLE 2.** Asymmetry perception survey results

	Percentage of faces correctly identified as symmetrical/asymmetrical (%)
0.5 mm survey	
Symmetrical (N = 135)	96.3 (n = 130)
Upper lid ptosis (N = 90)	18.9 (n = 17)
Upper lid retraction (N = 90)	6.7 (n = 6)
Lower lid retraction (N = 90)	18.9 (n = 17)
1 mm survey	
Symmetrical (N = 153)	93.5 (n = 143)
Upper lid ptosis (N = 102)	65.7 (n = 67)
Upper lid retraction (N = 102)	61.8 (n = 63)
Lower lid retraction (N = 102)	51.0 (n = 52)
1.5 mm survey	
Symmetrical (N = 153)	99.3 (n = 152)
Upper lid ptosis (N = 102)	92.2 (n = 94)
Upper lid retraction (N = 102)	73.5 (n = 75)
Lower lid retraction (N = 102)	73.5 (n = 75)
Inversion survey	
Symmetrical (N = 174)	90.8 (n = 158)
Inverted symmetrical (N = 174)	92.5 (n = 161)
Asymmetrical (N = 348)	80.7 (n = 281)
Inverted asymmetrical (N = 348)	61.0 (n = 212)

As expected, the percentage of symmetrical faces correctly identified as symmetrical was consistently high between the surveys (Tables 2 and 3). Each of the 3 asymmetries was found to be more detectable as they increased in size, with each being detected by most participants at 1 mm. The largest proportion of faces was correctly recognized at 1.5 mm (Tables 2 and 3). Combined, the layperson population detection of any asymmetry at 0.5 mm had a sensitivity of 42%, (95% CI: 37%–47%), at 1 mm the sensitivity rose to 71% (95% CI: 66%–75%), and at 1.5 mm sensitivity was 81% (95% CI: 76%–85%). Specificity was above 90% in all 3 surveys.

**TABLE 3.** 0.5 mm, 1 mm, and 1.5 mm survey results

	Percentage of faces correctly identified as symmetrical/asymmetrical (%)			*p* value	
	0.5 mm survey	1 mm survey	1.5 mm survey	0.5 mm vs. 1 mm survey	1 mm vs. 1.5 mm survey
Symmetrical	96.3	93.5	99.3	0.280	0.006
Upper lid ptosis	18.9	65.7	92.2	<0.001	<0.001
Upper lid retraction	6.7	61.8	73.5	<0.001	0.073
Lower lid retraction	18.9	51.0	73.5	<0.001	<0.001

The detection rates of asymmetry varied among the different faces in the study, but the differences were not statistically significant. The detection rate for face 1, face 2, and face 3 was 18.9%, 8.9%, and 16.7%, respectively, for the 0.5 mm survey (*p* = 0.140); 67.4%, 53.9%, and 56.9%, respectively, for the 1 mm survey (*p* = 0.110); and 85.3%, 74.5%, and 79.4%, respectively, for the 1.5 mm survey (*p* = 0.159).

Twenty-nine clinically associated participants completed an independent analysis, comparing ability to identify 1 mm asymmetries when presented in a normal or inverted orientation (Table 1). Of these participants, 19 were consultants (oculoplastic: n = 12; glaucoma: n = 3; pediatric ophthalmology: n = 2; vitreoretinal: n = 1; cornea: n = 1), 3 were specialty doctors, 5 were trainees, 1 was a fellow, and 1 was an associate specialist. Each participant scored 36 faces each, resulting in a total of 1,044 observations being counted (Table 2).

The analyses revealed that inversion did not significantly impact the ability to identify symmetrical faces when inverted (Table 4). However, a statistically significant difference was observed in the ability to recognize asymmetrical faces, with 80.7% correctly identified when presented in normal orientation compared with only 61.0% when the same images were presented in an inverted orientation, a drop of 19.7% (Table 4). This reflects a sensitivity of 81% (95% CI: 76%–85%) in normal orientation compared with 61% (95% CI: 56%–66%) when inverted.

**TABLE 4.** Inversion survey results

	Percentage of faces correctly identified as symmetrical/asymmetrical (%)	*p* value
Symmetrical	90.8	0.561
Inverted symmetrical	92.5	
Asymmetrical	80.7	<0.001
Inverted asymmetrical	61.0	

## DISCUSSION

The results of asymmetry surveys demonstrate that for the 3 eyelid asymmetries studied, upper lid ptosis, upper lid retraction, and lower lid retraction, a majority was detected at 1 mm. Correct detection of asymmetry increased as the size of the asymmetry increased. Upper lid ptosis was the most highly detectable at all severities. A proposed concept in facial asymmetry detection is that there is a “perceptive threshold,” where the asymmetry changes from being unnoticeable to abruptly become more detectable to observers.^6^ While this study only looked at 3 degrees of severity, a larger change was noted between the 0.5 mm and 1 mm surveys compared with the 1 mm and 1.5 mm surveys, which supports the “perceptive threshold” concept. However, as the asymmetries were not unnoticeable at the previous 0.5 mm severity, and continued to increase at the 1.5 mm severity, it could also suggest a more linear relationship between severity and detection. The reasons for the differences in the detection rates of asymmetry between the different faces, while not statistically significant, are likely to be multifactorial in nature. Future studies should ensure to include a greater number of participants, to include a wider range of face types and ethnicities, due to variability in detection across different faces.

While most asymmetries were correctly detected at 1 mm degree of severity, a minority was detected at 0.5 mm, and this may be viewed as unacceptable to some patients. The results presented here, we hope, will be useful for clinicians in providing informed counsel to patients on likelihood of laypersons to notice asymmetries and thereby aiding decision-making processes for whether intervention is appropriate.

The inversion survey results demonstrated a large drop in the correct identification of asymmetry in inverted images compared to images of normal orientation. This demonstrates that asymmetry perception is impaired by image inversion, a finding previously only demonstrated in terms of reduction in facial identification.^15–17^ This finding is important; the implication that surgeons performing surgery with an inverted intraoperative view may have an impaired asymmetry perception may result in decisions being made differently if the patient was observed with normal orientation. By making surgeons aware of this potential issue, a simple remedy would be to have the surgeon adjust their view in assessing eyelid asymmetry from an inverted surgeon’s view to a normal upright orientation to ensure more accurate intraoperative assessment of eyelid symmetry.

A limitation of the asymmetry surveys was the use of unnaturally perfectly symmetrical faces, which may have exaggerated the digitally manipulated asymmetries. Only the lid levels were manipulated, with crease height remaining unaltered. It is known that the apparent lid crease height may elevate with ptosis and lower with lid retraction, factors our model did not account for. While a variety of models of different genders were used, models were of a young age, which may have emphasized the asymmetries further. Participants were not restricted by time in viewing each face, so this would not replicate the situation of walking past someone on the street, instead being more like having a conversation with someone. Additionally, while the survey introductory instructions discussed facial asymmetry, context regarding the importance of assessing facial asymmetry with eyelid malposition surgery was mentioned, potentially biasing the participants to look at the eyelids. A limitation within the inversion survey was that surgeons in practice would see the patients at an angle instead of being directly above as produced in the face-on images. A further limitation within the inversion survey was that 17 of the 29 clinically associated participants were nonoculoplastic specialists who may not be accustomed to viewing inverted faces in the intraoperative view.

This study provides research to counsel patients with varying degrees of upper lid ptosis, upper lid retraction, and lower lid retraction, on how noticeable their eyelid asymmetry will be to laypeople. Image inversion decreases a clinician’s ability to detect asymmetry, indicating a surgeon performing surgery at the head of the bed should take action to remedy this, such as adjusting their view by walking around the bed and checking from the normal orientation.
